# Nanostructured ZnO-Based Electrochemical Sensor with Anionic Surfactant for the Electroanalysis of Trimethoprim

**DOI:** 10.3390/bioengineering9100521

**Published:** 2022-10-02

**Authors:** Vinoda B. Patil, Davalasab Ilager, Suresh M. Tuwar, Kunal Mondal, Nagaraj P. Shetti

**Affiliations:** 1 Department of Chemistry, Karnatak Science College, Dharwad 580001, Karnataka, India; 2 Department of Chemistry, K.L.E. Institute of Technology, Hubballi 580027, Karnataka, India; 3 Idaho National Laboratory, Idaho Falls, ID 83415, USA; 4 Department of Chemistry, School of Advanced Sciences, KLE Technological University, Vidyanagar, Hubballi 580031, Karnataka, India; 5 University Center for Research & Development (UCRD), Chandigarh University, Gharuan, Mohali 140413, Panjab, India

**Keywords:** trimethoprim, electrochemical sensor, zinc oxide nanoparticles, anionic surfactant, biomedical applications

## Abstract

In this research, detection of trimethoprim (TMP) was carried out using a nanostructured zinc oxide nanoparticle-modified carbon paste electrode (ZnO/CPE) with an anionic surfactant and sodium dodecyl sulphate (SDS) with the help of voltametric techniques. The electrochemical nature of TMP was studied in 0.2 M pH 3.0 phosphate-buffer solution (PBS). The developed electrode displayed the highest peak current compared to nascent CPE. Effects of variation in different parameters, such as pH, immersion time, scan rate, and concentration, were investigated. The electrode process of TMP was irreversible and diffusion controlled with two electrons transferred. The effective concentration range (8.0 × 10^−7^ M–1.0 × 10^−5^ M) of TMP was obtained by varying the concentration with a lower limit of detection obtained to be 2.58 × 10^−8^ M. In addition, this approach was effectively employed in the detection of TMP in pharmaceutical dosages and samples of urine with the excellent recovery data, suggesting the potency of the developed electrode in clinical and pharmaceutical sample analysis.

## 1. Introduction

Trimethoprim (TMP), chemically referred to as 5-(3,4,5-trimethoxybenzyl) pyrimidine-2,4-diamine, is used as an antibacterial medication to prevent and treat infections of the urinary, intestinal, middle ear travelers’ diarrhea, and respiratory tract [1]. In pharmaceutical manufacture, it is frequently used with sulfonamides (such as sulfamethoxazole) to induce synergistic effects [2]. TMP is also available in a single drug formulation. It is a synthetic antibiotic drug that prevents the formation of tetrahydro folic acid (a chemical required for bacteria and human cells to build proteins) by blocking the enzymes that convert dihydro folic acid into tetrahydrofolic acid. TMP hinders the bacterial enzyme higher than the human enzyme. Antibiotics, such as TMP, are given to animals and humans annually to cure and prevent illnesses and infections [3]. Many antibiotics are eliminated in conjugated forms that can be easily regenerated to their original condition and destroyed from dosed animals without metabolism. Antibiotics are referred to as “pseudo persistent” pollutants because they remain in the environment for a long time. As a result, antibiotics in the atmosphere have gotten a lot of attention. Additionally, this TMP leads to a very dangerously low level of thrombocytes by decreasing folic acid level and leads to the formation of bone marrow cells [4]. Hence, it is essential to propose a highly sensitive and selective technique to analyze TMP in biological and pharmaceutical samples.

In recent times, electrochemical sensors have piqued the interest of scientific researchers. Electrochemical sensors are used as sensitive detection techniques because of their fast response rate, better sensitivity, low cost, and easy fabrication [5,6]. In addition, electrochemical sensors are helpful in determining the biologically active molecule and gaining insight into its biochemical processes and a better knowledge of their interactions [7]. Carbon paste electrodes (CPEs) have gained a lot of consideration as a sensor material for a variety of reasons, including their chemical inertness, stability, robustness, minor ohmic resistance, lack of requirement for an internal solution, and are suitable in detection applications [8,9]. The CPEs are free from toxic, environmentally friendly electrodes. The passivation concerns are simply resolved throughout the scenario by a rapid and simple surface rejuvenation. However, the bare CPEs also exhibit some flaws in determination of electrochemical processes, including lesser sensitivity, slower electron transport, poor stability across a broad range of concentration solutions, and demand for a larger over-potential for the electro-catalytic technique. However, the electrodes’ modification can help avoid such problems [10,11,12]. The electron transport rate can be accelerated using chemically modified electrodes by lowering the over-potential. The nanoparticle (NP)-oriented chemical-modified electrodes are currently the focus of interest because of their intensified signal response, improved sensitivity, and higher stability.

ZnO nanoparticles comprise a wurtzite structure along with a wide surface area, making them an excellent biosensor material. At room temperature, the ZnO possesses a large band gap with a high excitation binding energy. ZnO nanoparticles’ chemical as well as physical properties differ from bulk ZnO nanoparticles. Electro and photoluminescent substances [13], gas sensors [14], solar cells, etc., are some examples of ZnO nanoparticle applications. The ZnO in powdered form shows excellent optical transmission and electrical conductivity. Furthermore, the electronegativities of the Zn and O atoms in ZnO nanoparticles range significantly, making the bonding extremely ionic. Most importantly, ZnO nanoparticles are less hazardous and cost efficient.

An anionic surfactant, sodium dodecyl sulphate (SDS), has a unique structure consisting of a lengthy hydrophobic tail on one side and a polar head on the other [15]. Adsorption of SDS on the sensing surface causes the accumulation of many negative ions on the electrode surface. Many works have reported the use of SDS in the field of electrochemistry [16,17]. As per the literature, some works are published regarding determination and detection of TMP, such as spectrophotometry [18,19,20,21,22,23,24], liquid chromatography [25,26,27,28], capillary electrophoresis [29], potentiometry [30], HPLC-UV [31], and HILIC-HPLC [32]. However, these methods are time consuming, need complexity in sample preparation, and are costly compared to the electrochemical techniques.

The literature survey reveals that no research was explored to investigate TMP employing ZnO/SDS/CPE electrodes. Furthermore, the modified electrode was easy to prepare, less costly, and gave stable results. Further, ZnO/SDS/CPE was highly sensitive and more selective. The electrochemical investigations were performed using voltametric strategies, such as CV and DPV. The observations were made by studying the different parameters, such as pH, scan rate, and concentration study, which suggested that the inclusion of SDS with ZnO nanoparticles increased the surface area and current response, thereby increasing the sensitivity with a low detection limit. Hence, this sensor was employed to determine TMP in tablets as well as urine samples and to study excipient interference; the findings revealed the relevance of the method in detecting TMP.

## 2. Experimental Section

### 2.1. Chemicals and Reagents

The TMP (98%) and SDS (99%) were acquired from Sigma-Aldrich (St. Louis, MO, USA) and utilized as received. Himedia chemicals Pvt. Ltd. (Mumbai, India) provided H_3_PO_4_ (98%), KH_2_PO_4_ (99%), and Na_2_HPO_4_ (98%), which were utilized for the preparation of electrolytic solution (0.2 M phosphate-buffer solutions) for various pHs (3.0–6.0). The required 0.5 mM stock solution of TMP was prepared in ethanol. The use of double-distilled water was maintained while performing all the investigations.

### 2.2. Instrumentation

The study of the electrochemical behavior of TMP was carried out by employing electrochemical workstation (CHI 1110C, USA) consisting of an electrochemical cell congaing three-electrode electrode system, namely platinum electrode (counter), Ag/AgCl (reference), and ZnO/SDS/CPE (working). To perform pH measurements Elico pH meter (Elico Ltd., Mumbai, India) was used. Nano surf (Switzerland) was used to take AFM measurements. JEOL (JSM-IT 500LA) (Japan) instrument was used for SEM analysis and for XRD diffraction studies, XRD instrument MODEL RIGAKU (Japan) was employed.

### 2.3. Preparation of Pharmaceutical Sample

Trimethoprim tablets (each having 200 mg TMP) were acquired from a nearby pharmacy. Using agitate and a mortar, tablets were finely grounded. Small portion of tablet powder equal to the TMP was dissolved in ethanol and diluted up to the 100 mL volumetric flask using double-distilled water, sonicated for 15 min. Under ideal experimental conditions, the tablet’s TMP (1.0 × 10^−5^ M) analysis was quantified using DPV and by employing calibration graph concentration of the drug.

### 2.4. Preparation of Urine Samples

The samples were acquired from healthy participants and stored in the refrigerator for later analysis. Samples were diluted with PBS of pH 3.0. A known amount of TMP was spiked to a sample and was subjected to DPV measurements. Then, the concentration of the TMP sample was estimated using a standard plot.

### 2.5. Synthesis of Nanostructure ZnO Nanoparticle

The precipitation processes synthesized ZnO nanoparticles using zinc nitrate and potassium hydroxide as precursors. Using double-distilled water, a 0.2 M concentration of zinc nitrate (Zn (NO_3_)_2_6H_2_O) and 0.4 M concentration of potassium hydroxide (KOH) solution were prepared. Later, potassium hydroxide was added slowly to the zinc nitrate solution with constant stirring. The white precipitate of ZnO was formed, centrifuged for 30 min in 5000 rpm, and cleaned with double-distilled water and ethanol to remove impurities; ultimately, the obtained product was calcined for 3 h at 500 °C [33].

### 2.6. Fabrication of Electrode

Using a mortar, the CPE was developed by combining graphite (70%) and paraffin oil (30%). Then, the mixed paste was filled into the Teflon tube of 3 mm diameter consisting of a copper rod for electrical conductance; the prepared electrode was polished on a smooth surface and cleaned with double-distilled water. After every determination, the paste was expelled and fresh paste was used for further analysis. ZnO/CPE was developed by adding 0.05 g of ZnO, 0.65 g of graphite powder and 0.3 mL of paraffin oil (05:65:30). Later, the different concentrations of SDS (0.05 mM–0.25 mM) were added to the test solution containing TMP and buffered solution. Maximum peak current was observed for the 0.1 mM SDS concentration (Figure S1). Hence, further studies were carried out in this optimal condition. 

### 2.7. Experimental Procedure 

In the activation of the developed electrode, pH 3.0 was employed for the electrochemical investigation of TMP. Stock solution of TMP was prepared with the help of ethanol. The electrochemical investigation and impact of pH, accumulation time, surfactant concentration, and scan rate variation were studied by CV technique under optimized conditions. The effects of concentration, pharmaceutical sample analysis, urine sample analysis, and interference study were examined utilizing differential pulse voltammetry in a potential range 0.8–1.4 V, 3.0 pH at an accumulation time of 10 s.

## 3. Results and Discussion

### 3.1. Surface Area of Electrode and ZnO Characterization

**Figure 1:**
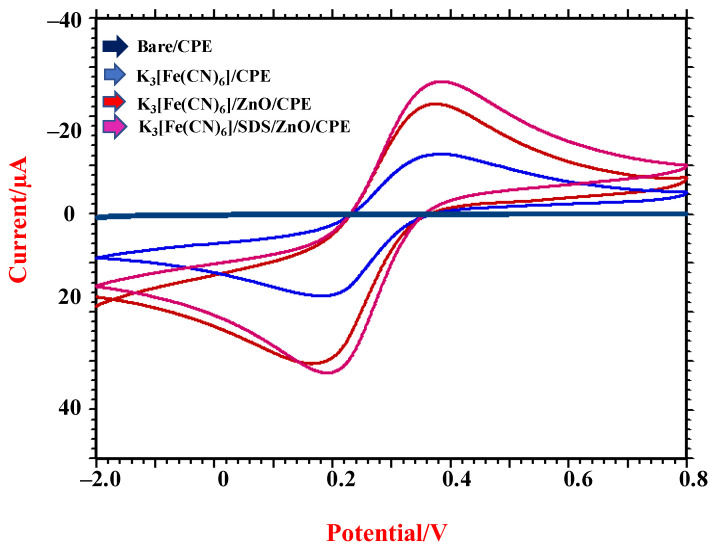
CV measurements of 1.0 mM K_3_(Fe (CN)6) at bare CPE and ZnO/CPE and ZnO/SDS/CPE.

To calculate the active area of the developed sensor, 1.0 mM K_3_(Fe (CN)_6_) was used as a probe and 0.1 M KCl as a supporting buffer. The CV measurements were taken for various scan rates displayed in Figure 1. Randle Sevcik Equation (1) was employed in calculating the area of the electrode [34] with the help of the value of slope obtained from Ip vs. √ υ plot. We calculated the electrode’s area, which was found to be 0.058 cm^2^, 0.094 cm^2^, and 0.099 cm^2^ for bare CPE, ZnO/CPE, and ZnO/SDS/CPE, respectively.

(1) $$\;\mathrm{Ip}=2.69\times 10^{5}\times n^{3/2}\times A\;\times D0^{1/2}\times \upsilon^{1/2}\times C_{o}$$

### 3.2. Characterisation of Modifier

**Figure 2:**
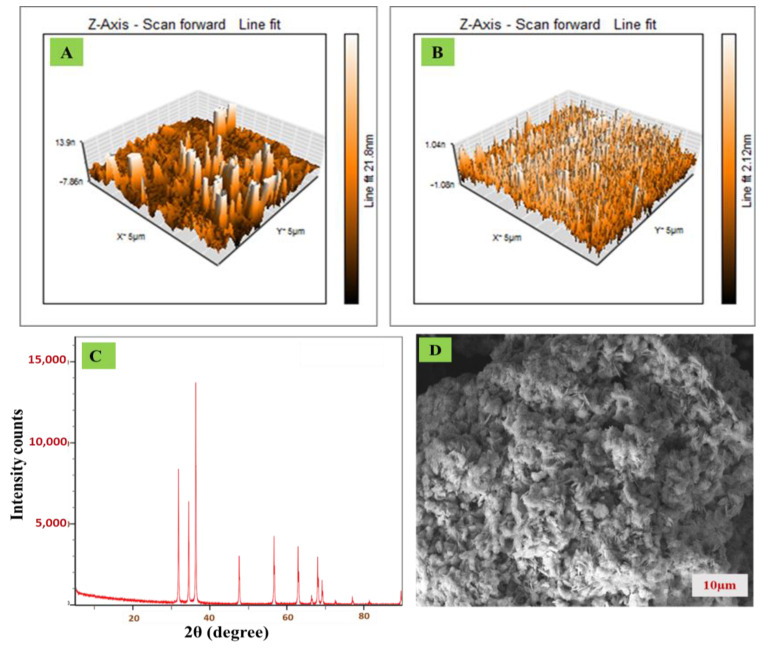
Characterization of ZnO nanoparticles (**A**) AFM of CPE; (**B**) AFM of ZnO/CPE; (**C**) XRD of ZnO; (**D**) SEM morphology of ZnO.

Atomic force microscopy (AFM), scanning electron microscopy (SEM), and X-ray diffraction (XRD) studies were carried out to understand the morphological characteristics of the sensing surface. AFM images of bare and ZnO/CPE are displayed in Figure 2A,B. The total area roughness (Z) for bare and ZnO/CPE was found to be 0.4 and 1.12 nm. The XRD pattern of ZnO reveals firm diffraction peaks, indicating the sample’s crystalline composition. The diffraction peaks were intense at 2θ° = 32.8, 34.4, and 36.2. However, the diffraction peaks further appeared at 47.5, 56.6, 62.8, 66.3, and 67.9, which were due to the various diffraction phases of ZnO exhibiting the hexagonal wurtzite structure. The absence of impurities in diffraction patterns revealed that the prepared ZnO is of high purity (Figure 2C). Further, SEM analysis showed that synthesized ZnO has an irregular shape and size with large surface area, which is depicted in the SEM image in Figure 2D.

### 3.3. Electrochemical Detection of TMP

**Figure 3:**
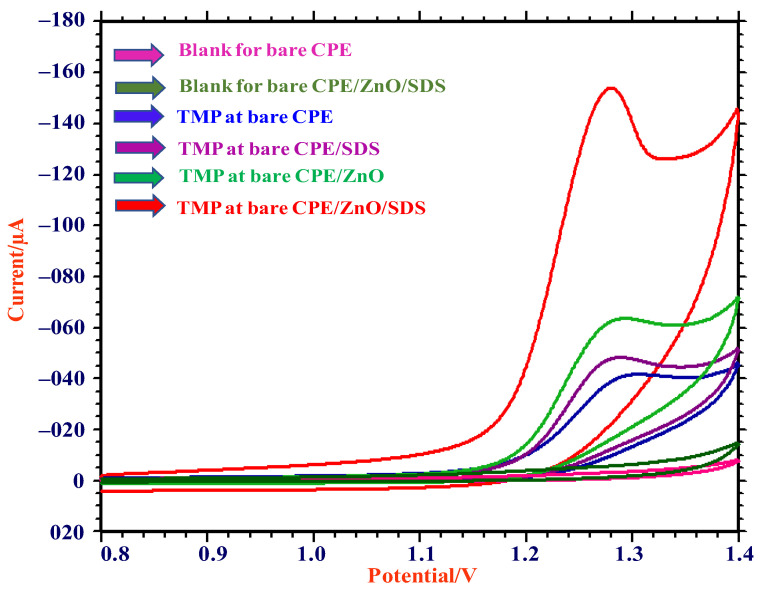
CV responses of 0.5 mM TMP in pH 3.0 (0.2 M PBS) at CPE, SDS/CPE, ZnO/CPE, and ZnO/SDS/CPE.

Electrochemical investigations of TMP were performed by employing the CV technique using 0.5 mM of TMP (in pH 3.0) at scan rate 0.05 V/s. Figure 3 shows the presence of an oxidation peak for all four electrodes (CPE, SDS/CPE, ZnO/CPE, ZnO/SDS-CPE). However, the double-fold-enhanced peak intensity was observed for ZnO/SDS-CPE at 153.9 µA, with a peak potential value of 1.280 V. In the presence of SDS, a good interaction occurred between the negatively charged monomers of SDS and they were strongly attracted towards the positively charged amine moiety on TMP (Scheme S1). On the reverse scan, the reduction peak was not detected and it indicates the process to be irreversible.

### 3.4. Immersion Time

**Figure 4:**
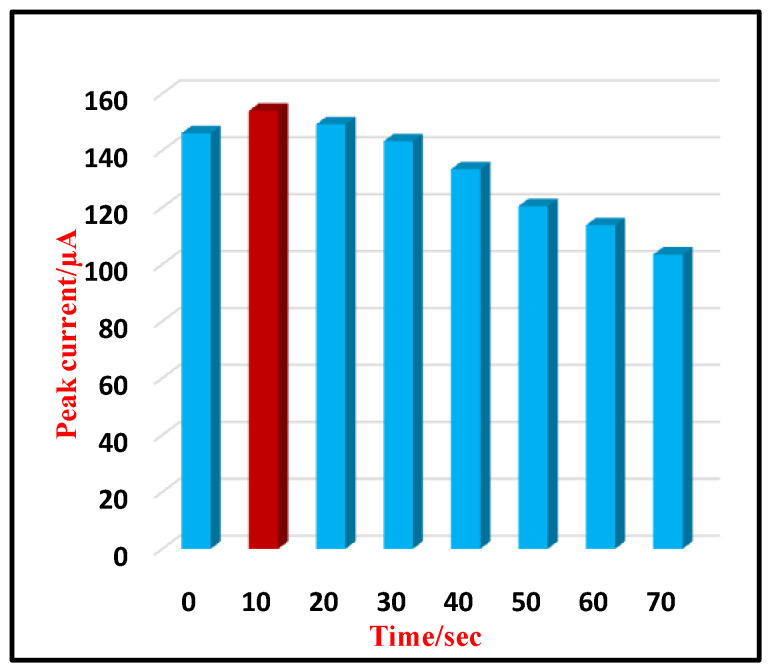
Plot of Ip and immersion time (t_imm_) for 0.5 mM TMP.

The time required for an analyte molecule to accumulate at the electrode surface can influence the electrode’s sensitivity. Hence, to study the effect of accumulation time, CV measurements were recorded for different time intervals from 0 to 70 s using 0.5 mM TMP at 0.05 V/s scan rate. The change in peak intensity was observed with respect to time and higher peak intensity was obtained at 10 s (Figure 4) and, hence, 10 s was preferred as an immersion time for further investigations.

### 3.5. pH Study

**Figure 5:**
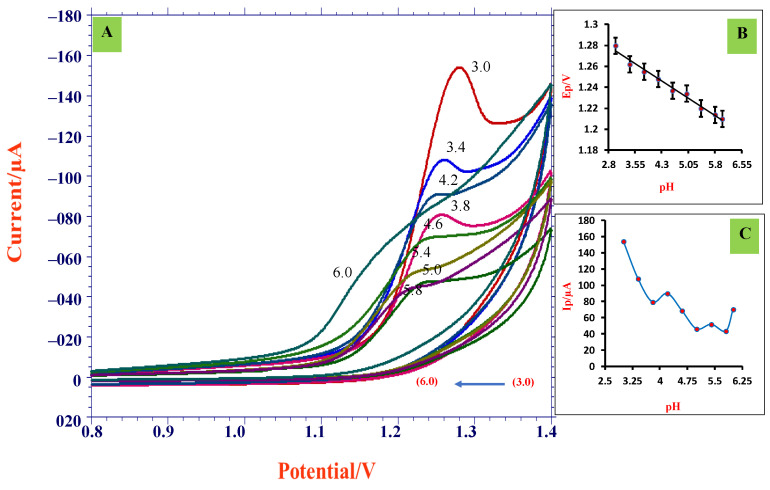
(**A**) CVs obtained for 0.5 mM TMP for various pH (pH 3.0–pH 6.0) employing ZnO/SDS/CPE at scan rate 0.05 V/s; (**B**) dependence of potential (Ep) on pH; (**C**) plot of peak current (Ip) vs. pH.

An electrochemical examination associates the control of peak potential by utilizing different supporting electrolytes. This was executed to enhance the conductivity of the solution. We initially examined the electrochemical behavior of TMP at ZnO/SDS/CPE in different electrolytic solutions, such as citrate buffer, Briton-Robinson buffer, sulphuric acid, and PBS at pH 3.0 (Figure S2). A well-defined oxidation peak was observed in the PBS-supporting electrolyte. Impact of PBS was studied for 0.5 mM TMP at ZnO/SDS/CPE for different pH ranges (from 3.0 to 6.0) using the CV technique at 0.8–1.4 V potential window. Voltammograms are displayed in Figure 5A. A well-resolved single oxidation peak was detected for all pH ranges and it can be noticed that with the rise in pH of supporting buffer concentration, a shift in peak potential (Ep) occurred, which is towards the less positive side, revealing the presence of H^+^ in the oxidation of TMP [35]. After investigating the pH range above 6.0, we noticed the absence of a peak current, which concludes that the oxidation of TMP at ZnO/SDS/CPE can be acidic. The graphs for Ep and pH are plotted in Figure 5B, which gave a fitted equation Ep = −0.0219 pH + 1.340, R^2^ = 0.988 and the slope value obtained was −0.0219 V/pH, which is nearest to the standard value of −0.021 V, which specifies that the electron and proton engaging in the reaction mechanism are uneven in number [36]. From Figure 5C, we can observe that at pH 3.0, the peak current (Ip) was intense; hence, the supporting electrolytic solution of pH 3.0 was chosen for further measurements. Further, we examined the impact of pH using the DPV technique and obtained results similar to the above CV results (Figure S3). 

### 3.6. Scan Rate Study

**Figure 6:**
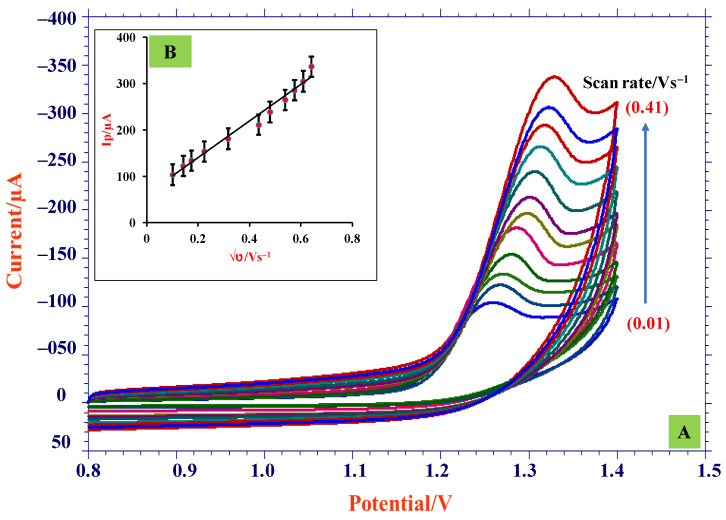

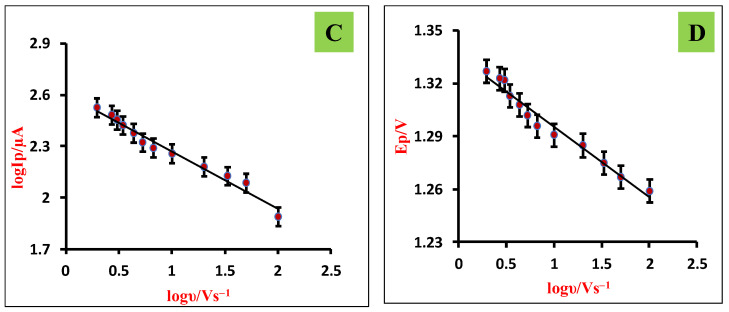
(**A**) CVs obtained for various scan rates (0.01–0.41 V/s); (**B**) dependence of Ip and √υ; (**C**) dependence of log Ip and log υ; (**D**) plot of Ep vs. log υ.

The scan rate studies give insights into reaction mechanisms and the dependence of peak current and peak potential on scan rate. Therefore, the impact of scan rate on electro-oxidation of TMP was studied by employing CV technique at ZnO/SDS/CPE using 0.5 mM TMP by altering the scan rate from 0.01 to 0.41 V/s with its optimum pH at 3.0 (Figure 6A). The voltammograms showed that with a peak current increase, peak potential slightly shifted towards the positive direction and peak current increased gradually, indicating the irreversible nature of reaction [37]. The increase in peak intensity with increasing scan frequency is due to the signal being anticipated due to improved charge transfer during system capacitance loading. In addition, a linear equation for the log of Ip vs. log ν (Figure 6C) acquired is shown below.

log Ip = −0.384 log ν + 2.604; R^2^ = 0.978

Resultant slope (−0.384) was significantly closer to the predicted value 0.5; therefore, we can conclude that the reaction was governed by the process of diffusion [38]. From plot, Ip vs. √ν (Figure 6B) gave the linearity equation below.

Ip = 393.21√ν + 61.575; R^2^ = 0.980

Ep = −0.0402 logν + 1.356; R^2^ = 0.969

The linear relationship was obtained from the plot peak potential (Ep) vs. log scan rate (log ν) (Figure 6D) and the intercept of this plot was substituted in the Laviron Equations (2) and (3), which were employed to measure the transfer coefficient (α) and heterogeneous rate constant (k°), respectively.

Further, the number of electrons was estimated to be two (2.42) [39].

(2) $$\mathrm{Ep}-\mathrm{Ep}/2=\left(\frac{47.7}{\alpha }\right)\mathrm{mV}\;$$

(3) $$E=E_{o}+\left(\frac{2.303\mathrm{RT}}{\left(1-\alpha \right)\mathrm{nF}}\right)\log\left(\frac{\left(1-\alpha \right)\mathrm{nF}}{\mathrm{RTk}{}^{\circ}}\right)+\left(\frac{2.303\mathrm{RT}}{\left(1-\alpha \right)\mathrm{nF}}\right)\log\left(ט\right)$$

By calculation, the electrons (α) included in the oxidation of TMP were found to be 0.58 and k° value obtained was 1.28 s^−1^.

Using the equations below, (4) and (5), the number of proton transfer and surface coverage concentration (Γ*) were calculated to be 1(0.89) and 7.58 × 10^−6^ mol cm^−2^, respectively.

(4) $$\frac{\mathrm{dEp}}{\mathrm{dpH}}=\frac{2.303\mathrm{RTm}}{\mathrm{nF}}$$

(5) $$\mathrm{Ip}=\frac{n^{2}F^{2}A\;n\;\Gamma *\;\nu }{4\;R\;T}\;$$

### 3.7. Possible Electrode Mechanism

**Scheme 1:**
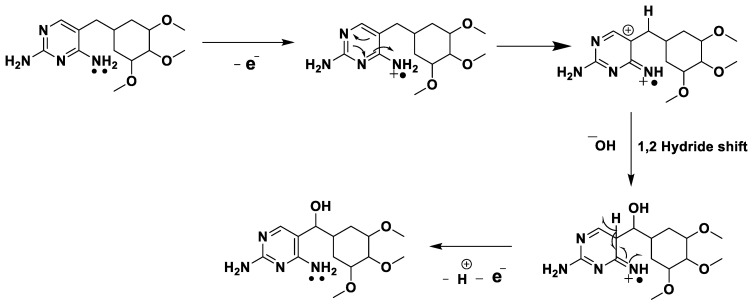
Probable electrochemical mechanism of TMP at ZnO/SDS/CPE.

In the electrochemical reaction of TMP, pH investigation confirmed the presence of protons, whereas per variation of scan rate studies, two electrons were transferred in the electrochemical oxidation reaction. Further, in this mechanism, electron losses occur initially from a non-bonding electron from nitrogen (non-bonding electrons are higher in energy). The electron-deficient nitrogen pulls the electron from the pyrimidine ring. As a result, positive charge was created and an unstable ion is formed. Hence, a rapid 1,2 hydride shift takes place, leading to the formation of secondary carbocation, which is attacked by the hydroxide ion. From the collected data, the plausible electrochemical mechanism can be predicted, as described in Scheme 1. 

## 4. Analytical Applications

### 4.1. Variation in TMP Concentration

**Figure 7:**
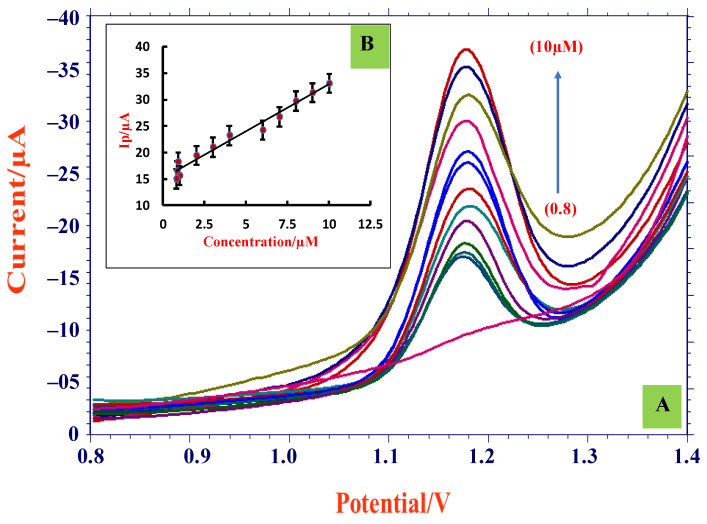
(**A**) DPVs of concentration variation (0.8 µM–10 µM) at ZnO/SDS/CPE; (**B**) graph of Ip and concentration.

DPV approach was opted for concentration variation studies as the CV technique was not sensitive in detecting TMP in lower concentrations. Variation in concentration was observed using the developed electrode at its optimum pH 3.0. From Figure 7A, it can be noticed that with a decrease in TMP concentration, the peak current declined and the linearity range obtained was 8.0 × 10^−7^ M–1.0 × 10^−5^ M. By using the intercept and slope values acquired from plot of Ip and concentration (Figure 7B), the limit of detection and limit of quantification were measured employing the below equations [40]:

(6)    LOD=3sm

(7) LOQ=10s⁄m 

The formula ‘s’ is referred to as standard deviation of intercept value and ‘m’ corresponds to mean of the slope, which was calculated for three sets of measurements and the LOD and LOQ calculated data obtained were 2.58 × 10^−8^ M and 8.61 × 10^−8^ M, respectively. The obtained LOD value was compared with other reported methods (Table 1) and it was found that the present method was sensitive with a lower detection limit because of the novel properties of zinc oxide (semiconductor material with large excitation binding energy of 60 meV). Furthermore, ZnO is a favorable material for sensor applications due to its high catalytic ability, extraordinary electrical conductance, nontoxicity, and chemical stability. The resultant LOD and LOQ values showed that the developed electrode was sensitive, convenient, and efficient in TMP sensing and detection. 

### 4.2. Pharmaceutical Sample Analysis

A pharmaceutical dosage of TMP was obtained from a local medical shop with the brand name Bacstol 200 mg. The tablet was crushed using a mortar and then the tablet powder was dissolved in ethanol and diluted up to the 100 mL volumetric flask, followed by sonication for a few minutes. Then, the solution was filtered to eliminate the undissolved solute. The filtrate obtained was subjected to DPV measurements, shown in Figure S4; the concentration was compared with the help of a calibration graph. The concentration and the recovery achieved are represented in Table 2.

### 4.3. Analysis of Urine Sample

The proposed method was validated by analyzing the urine samples obtained from healthy people. The sample was diluted to using a supporting electrolyte of pH 3.0. The sample was then filtered and the supernatant solution was spiked with a known amount of TMP and analyzed using the DPV technique (Figure S5). The use of a calibration graph calculated the recovery range. As such, 97.5–99% recovery was obtained with RSD 1.62%. Table 3 provides details about analyzed data.

### 4.4. Effect of Interference Molecules

The excipient study was conducted to check the interference of some of the commonly used excipients, such as citric acid, lactose, dextrose, sucrose, glycine, glucose, urea, sodium chloride, potassium chloride, calcium chloride, and potassium sulphate. The concentration of 0.01 mM excipient solution was chosen and investigated. The changes in peak potential were observed to a smaller extent but were less than ±5% (Table 4), which confirmed that the excipients do not intervene much in TMP detection.

### 4.5. Repeatability and Reproducibility

To verify the functionality and viability of ZnO/SDS/CPE, inter-day and intra-day measurements were performed. Three replicates of measurements for 0.01 mM TMP were taken on the same day to study the repeatability nature of an electrode and the electrode retained its initial peak intensity, with 99% recovery, shown in Figure S6. To examine the reproducibility nature of an established electrode, it was kept in a dark place in an airtight jar and DPVs were recorded for 0.01 mM TMP. The results showed 97% recovery with an RSD value of 0.81%, which verified the efficiency of the sensor.

## 5. Conclusions

In this research, TMP was determined by electrochemical techniques, such as CV and DPV, at ZnO/CPE along with anionic surfactant SDS. The enhanced peak current for TMP was noticed at the proposed sensor in its optimum pH 3.0 of PBS. We performed scan rate investigations to gather information about reaction kinetics. The study suggested the process to be diffusion controlled and a total of two electrons was transferred during the reaction process, along with a single proton. Concentration variation studies observed the linear relationship between peak current and TMP concentration. Detection limit was calculated (2.58 × 10^−8^ M) and compared with other reported methods. Excipient investigations verified that they did not interfere with the excipients in signal response of TMP. Overall, the developed electrode was easy to fabricate, was cost effective, and could produce reproducible results. Furthermore, ZnO/SDS/CPE provides the possibility of a miniaturized system, offering high sensitivity and a quick response with a small amount of analyte. Hence, the developed method can be applied in the determination of clinical samples in trace quantities.

## Figures and Tables

**Table 1:** Comparative study of detection limit of TMP.

Electrode Used	Technique Used	Linearity Range	LOD	References
Boron-doped diamond electrode	HPLC	85–1300 µM	5.5 µM	[24]
Methacrylic acid/molecularly imprinted polymer sensor	Potentiometry	0.03–26 µM	0.4 µM	[26]
molecularly imprinted polymer-graphene/Glassy carbon electrode	CV	1.0–100 µM	0.1 µM	[41]
Silver nanoparticle-graphene/Glassy carbon electrode	DPV	1.0–10 µM	0.4 µM	[42]
Graphene-Zinc oxide/Glassy carbon electrode	DPV	1–180 µM	0.3 µM	[43]
Cetyl trimethylammonium bromide/carbon paste electrode	CV	0.2–1.0 µM	0.1 µM	[44]
Printex L6 carbon black/copper (II) phthalocyanine film/Glassy carbon electrode	SWV	0.4–1.1 µM	0.6 µM	[45]
Multi-walled carbon nanotubes modified with antimony nanoparticles/CPE	DPV	0.1–0.7 µM	0.031 µM	[46]
ZnO/SDS/CPE	DPV	0.8–10 µM	0.025 µM	Present method

**Table 2:** Tablet analysis of TMP.

TMP	Observations
Labelled claim (mg)	200
Obtained amount(mg)	198
RSD %	1.06
Added (mg)	1.0
Obtained (mg)	0.99
Recovery %	99.36

**Table 3:** Urine sample analysis of TMP.

TMP Sample	Added (×10^−5^ M)	Obtained	Recovery (%)	RSD%
1	1.0	0.99	99	0.90
2	0.8	0.78	97.5	2.11
3	0.6	0.59	98.33	1.85

**Table 4:** Excipient study of TMP.

Excipients	Potential Observed (E_p_)	Change in Signal (%)
Citric acid	1.190	1.01
Lactose	1.188	0.84
Dextrose	1.181	0.59
Sucrose	1.191	1.10
Glycine	1.184	0.50
Glucose	1.190	1.01
Urea	1.174	−0.33
Salts		
Potassium chloride	1.171	−0.59
Sodium chloride	1.163	−1.27
Calcium chloride	1.183	0.50
Potassium sulphate	1.192	1.18

## Data Availability

The original data were available from the corresponding author upon an appropriate request.

## Supplementary Materials

The following supporting information can be downloaded at: https://www.mdpi.com/article/10.3390/bioengineering9100521/s1, Figure S1: Optimaiztion of SDS concentration; Figure S2: impact of different buffers on TMP; Figure S3: Effect of pH on behavior of TMP by DPV method; Figure S4: DPV for tablet analysis; Figure S5: DPV for urine samples; Figure S6: Reapatibility of SDS/ZnO/CPE sensor at 0.01 mM TMP; Scheme S1: Probable interaction of SDS and TMP.
