# Supplementary material for: Nanostructured ZnO-Based Electrochemical Sensor with Anionic Surfactant for the Electroanalysis of Trimethoprim
Source: Bioengineering (Basel). 2022 Oct 2;9(10):521. doi: 10.3390/bioengineering9100521 (PMC9598839; doi:10.3390/bioengineering9100521)
Supplement: Supplementary file 1 [file bioengineering-09-00521-s001.zip › bioengineering-1936077-supplementary.pdf]

SUPPORTING INFORMATION

Nanostructured ZnO based electrochemical sensor with anionic surfactant for the electroanalysis of trimethoprim

Vinoda B. Patil[1], Davalasab Ilager[2], Suresh M. Tuwar[1], Nagaraj P. Shetti[3*]

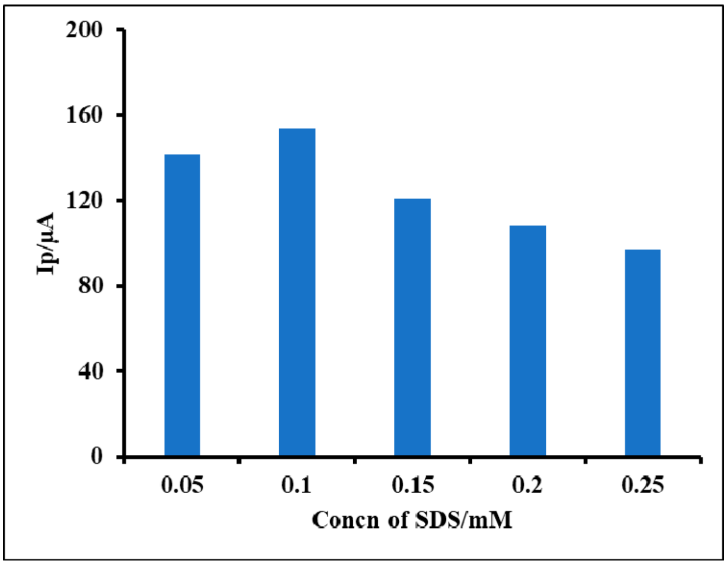

Figure S1. Different concentration of SDS (0.05mM,0.1mM, 0.15mM, 0.2mM, 0.25Mm) at 0.05 mM TMP with scan rate 0.05V/s.

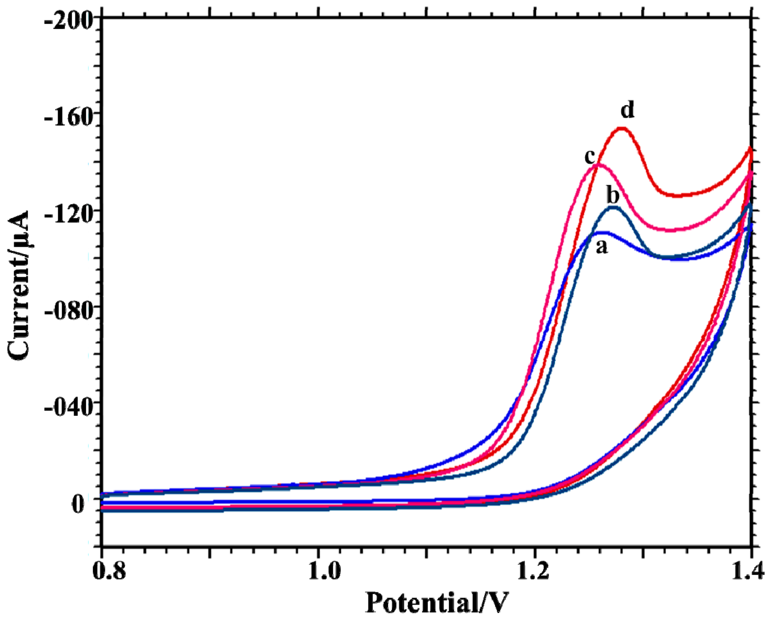

Figure S2. CV Voltammogram of different buffers (a; citrate buffer, b; sulphuric acid, c; Briton-Robinson buffer, d; phosphate buffer solutions) at 0.05 mM TMP with scan rate 0.05V/s.

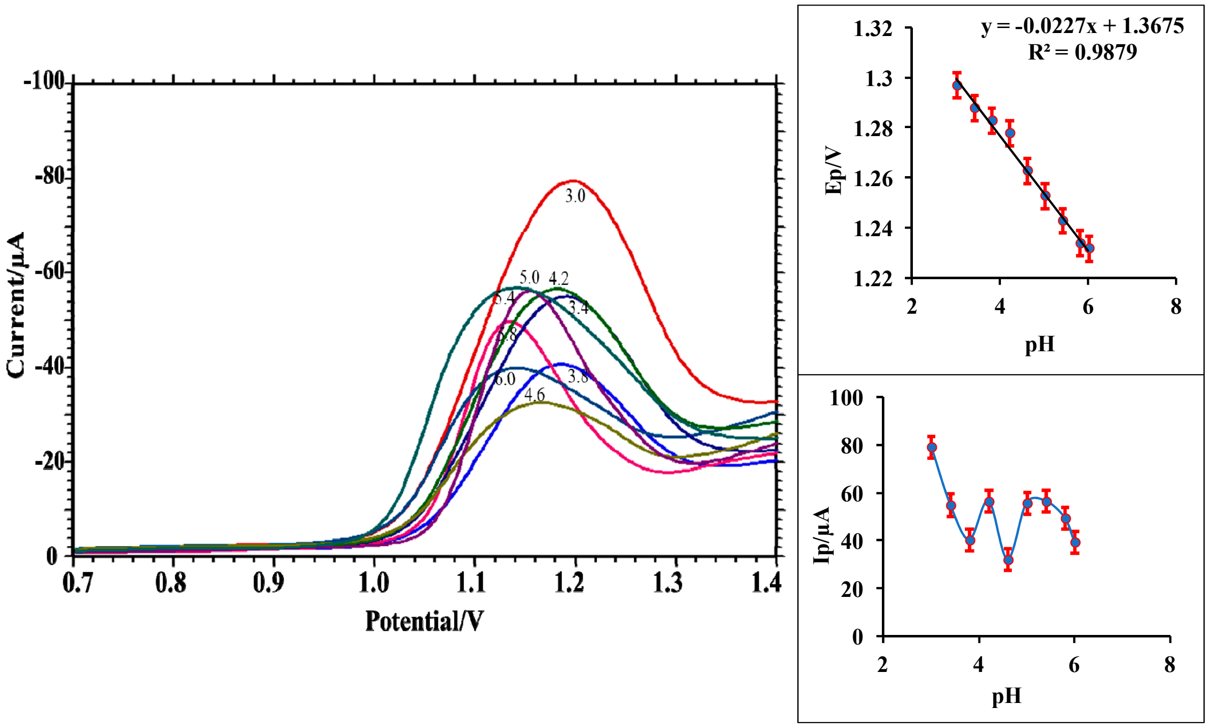

Figure S3. Effect of pH on electrochemical behavior of TMP by DPV method.

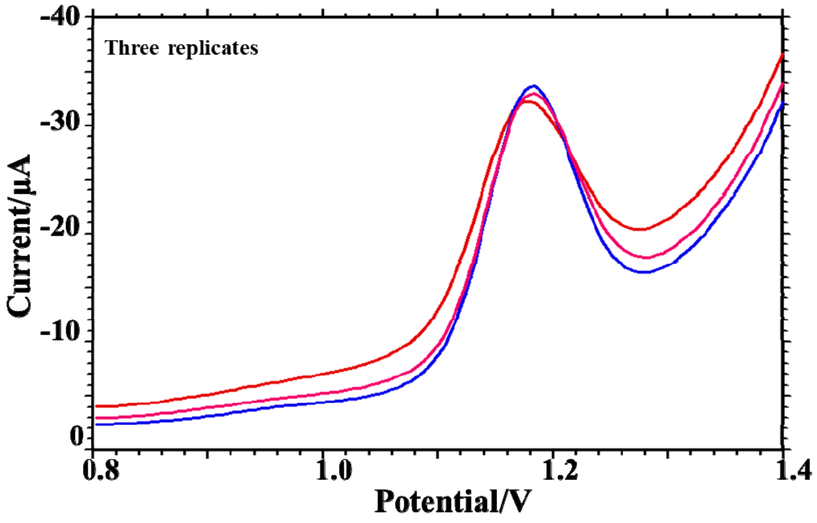

Figure S4. DPV for tablet analysis.

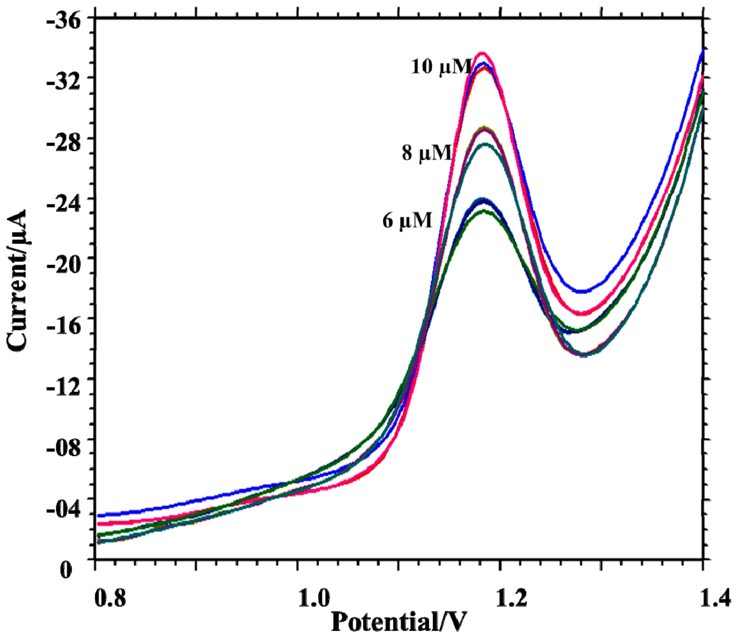

Figure S5. DPV for urine samples

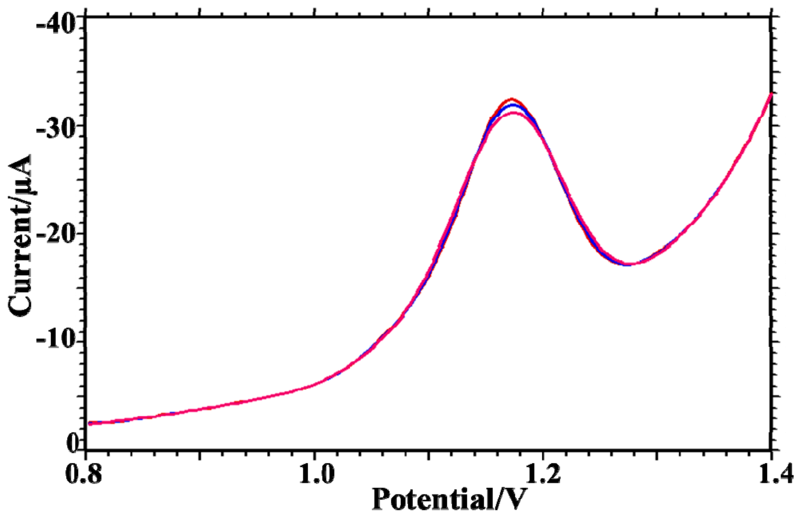

Figure S6. Reapatibility of SDS/ZnO/CPE sensor at 0.01mM TMP.

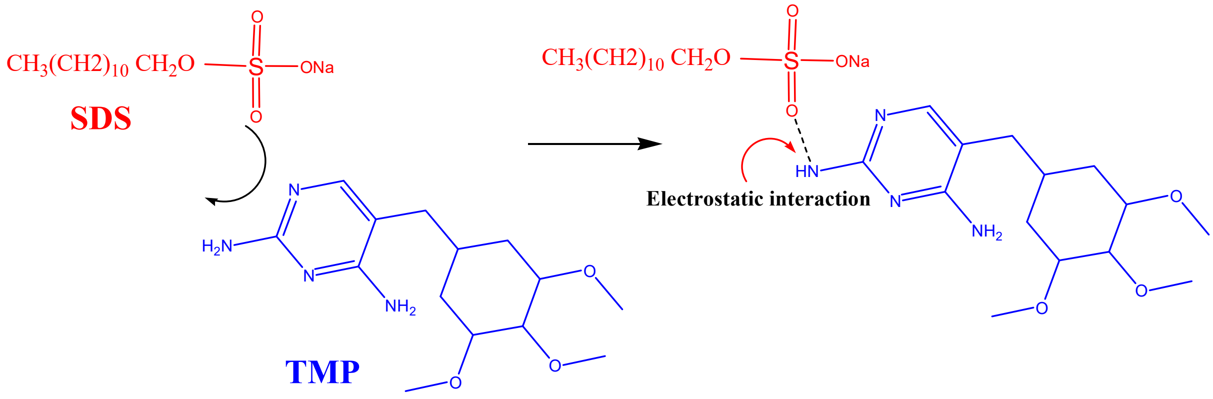

Scheme S1**.** Probable interaction of SDS and TMP.
